# Post-traumatic growth, resilience, perceived social support, and coping style among parents of very low birth weight infants: a multi-center, cross-sectional study

**DOI:** 10.3389/fpubh.2025.1686820

**Published:** 2025-12-04

**Authors:** Lily Wu, Yuqing Pan, Xiu-Min Jiang

**Affiliations:** 1 School of Nursing, Fujian Medical University, Fuzhou, China; 2 Fujian Maternity and Child Health Hospital, College of Clinical Medicine for Obstetrics & Gynecology and Pediatrics, Fujian Medical University, Fuzhou, China; 3 Fujian Obstetrics and Gynecology Hospital, Fuzhou, China

**Keywords:** infant, very low birth weight, post-traumatic growth, resilience, perceived social support, coping style

## Abstract

**Background:**

Post-traumatic growth (PTG) can guide parents to cherish life more deeply, thereby improving the quality of care they provide to their children. However, despite evidence linking a psychological resilience with PTG, the processes through which aspects of resilience influences PTG remain unclear. This study investigated the mediating role of perceived social support and coping styles in the relationship between resilience and PTG.

**Methods:**

In this cross-sectional study, 344 parents of very low birth weight (VLBW) infants admitted to the neonatal intensive care units of nine hospitals at level two or above in Fujian Province were selected by convenience sampling. Five self-reported questionnaires were completed by the participants. The data were analyzed using SAS 9.4. Structural equation modeling was employed to determine the relationships between the components using AMOS 24.0.

**Results:**

Psychological resilience can directly influence PTG (effect = 1.598, 95% CI = 1.388–1.824, *p* < 0.05) or indirectly affect it through the mediating role of positive coping style (effect = 0.039, 95% CI = 0.009–0.079, *p* < 0.05). Meanwhile, perceived social support can only influence PTG through a positive coping style (effect = 0.020, 95% CI = 0.004–0.047, *p* < 0.05).

**Conclusion:**

This study provides further insight into the importance of strengthening resilience, providing support, and developing positive coping strategies from nurses and healthcare providers for promoting PTG among VLBW infants’ parents. It is especially important to implement interventions directly targeting the enhancement of resilience among parents of VLBW infants.

## 1 Introduction

Very low birth weight (VLBW) infants, defined as neonates with a birth weight under 1,500 g (1), represent approximately 1.4% of all newborns (2) and are at a higher risk of neonatal mortality due to small gestational age (3). Advances in prenatal and neonatal intensive care have led to the increased admission of VLBW infants into neonatal intensive care units (NICUs) for life-saving treatments, such as continuous cardio-respiratory monitoring and advanced life support (4). Parents of these infants often experience various forms of stress, including a lack of knowledge about their child’s condition, concerns over treatment efficacy, limitations in parental roles, and the significant burden of NICU hospitalization, all of which contribute to potential trauma experienced during this challenging period (5).

However, research has also shown that some parents undergo beneficial psychological changes following traumatic events (6, 7). Post-traumatic growth (PTG)—characterized by an enhanced appreciation for life, improved relationships, increased personal strength, identification of new possibilities, and spiritual growth—is closely associated with preceding periods of anxiety, depression, and feelings of helplessness (8, 9). Parents with higher levels of PTG are more likely to engage positively with their psychological experiences and exhibit better treatment compliance (9). PTG can also stimulate parents to cherish life more deeply, adopt healthier coping strategies, and utilize available social resources to address problems, thus improving the quality of care they provide to their children (7). Parents of VLBW newborns experience significant psychological distress in the months following birth, facing increased mental health issues, parenting stress, and negative impacts on family dynamics compared with parents of full-term, normal birth weight children (10, 11). Even the hospitalization of the infant can be considered a stressful or traumatic event (4). Focusing on PTG levels during the hospitalization of VLBW infants is crucial for enhancing parental care quality and alleviating their stress.

Scholars are continuously refining the theoretical model of PTG (9), emphasizing individual characteristics such as personal traits and psychological resilience, the extent of psychological distress experienced, and cognitive processes in the development of PTG. Resilience, which belongs to psychological factors or personal traits, can be defined as the ability to the ability to survive and recover from adversity (12, 13). Individuals with strong psychological resilience are able to draw upon their internal resources and seek help from family, society, and other external sources to alleviate stress, maintain mental health, and improve their living conditions, thus achieving higher levels of PTG (14).

Another important variable is social support. Perceived social support is the help believed to come from social ties with other individuals, groups, and the larger community (15, 16). It can positively predict PTG levels and serve as a vital source of assistance for individuals facing stressful situations, helping mitigate stress responses (17). The relationship between perceived social support and PTG, however, may be more complex than it appears (18, 19). Individuals with optimistic open personality traits are able to elicit and accept social support more easily, which in turn promotes their pursuit of benefits and meaning construction, resulting in intentional reflection and enhanced feelings of hope.

Some researchers have found that coping strategies are related to the development of PTG. Lazarus et al. (19) proposed the stress–coping–adaptation(SCA) model, suggesting that as individuals confront stressors, their cognitive appraisal of the situation generates evaluative outcomes, which subsequently activate response resources and lead to coping outcomes. Coping outcomes affect individuals’ life concept, adaptability, and physical and mental health. Coping here refers to the conscious, purposeful, and flexible behavioral methods, means, and strategies that individuals adopt in response to environmental changes (20). These include both positive coping styles (e.g., seeking support and changing one’s value systems) and negative ones (e.g., avoidance and venting). Positive coping styles are a key component in achieving PTG (16). Consequently, task-oriented coping—a typical type of active coping— entails a focus on problem-solving, in contrast to avoidance, fantasy, and other strategies (21).

Based on previous studies, both the SCA and the PTG models can serve as theoretical frameworks for understanding the interrelationship between resilience, perceived social support, coping styles, and PTG in parents of NICU-hospitalized VLBW infants. When faced with the challenging circumstances of their VLBW infant’s premature birth and subsequent NICU admission, parents assess and respond to stress based on their personal traits and available environmental resources, employing unique coping styles to navigate these challenges. We assume that parents’ resilience, as a personal resource, and their perceived social support will enhance their positive coping styles, ultimately fostering PTG. Accordingly, this study proposes three hypotheses:

*H1*: Psychological resilience, perceived social support, and positive coping styles all positively affect the PTG of parents of VLBW infants.

*H2*: Psychological resilience can directly affect PTG and can also indirectly affect PTG through the mediating roles of perceived social support and positive coping, respectively.

*H3*: Perceived social support and positive coping styles act as chain mediators between psychological resilience and PTG.

## 2 Methods

This study was performed in adherence to the Strengthening the Reporting of Observational Studies in Epidemiology (STROBE) guidelines (22).

### 2.1 Study design and participants

The study employed a multi-center, cross-sectional design, conducted across nine comprehensive or specialized hospitals in Fujian Province from January 2022 to December 2022. Participants were recruited from the NICU using convenience sampling. The inclusion criteria were as follows: (1) all participants were parents with infants diagnosed as VLBW, with a gestational age of less than 37 weeks, and admitted to the NICU after birth; (2) the duration of hospitalization for the newborn was between 4–6 weeks; (3) parents were 18 years of age or older; and (4) participants were capable of verbal communication. Exclusion criteria included (1) parents who had experienced other adverse life events in the past 3 months, such as bereavement, a car accident, or the diagnosis of a serious illness (e.g., cancer); (2) parents who discontinued treatment or transferred their children to other hospitals due to disease progression; and (3) parents diagnosed with a mental illness.

### 2.2 Sample size calculation

The sample size was determined based on a prior survey, with PTG as the primary outcome measure. Assuming the mean and standard deviation of PTG scores were 53.60 and 12.14, respectively, and a delta of 1.32 with an alpha of 0.05, 325 participants were deemed necessary. Accounting for a 10% non-response rate, the minimum sample size was calculated to be 358. A total of 360 participants were enrolled in the study.

### 2.3 Measures

#### 2.3.1 General characteristics

A self-designed questionnaire was used to capture the general demographic characteristics of maternal pregnancy-related information and children’s hospitalization-related details (Additional file 1). This included data on age, education, residence, occupation, monthly income per capita, religious beliefs, and relationship with the child. Maternal pregnancy-related information encompassed parity, pregnancy complications, number of fetuses, delivery mode, and mode of conception. Hospitalization-related information included the child’s gender, gestational age, birth weight, whether mechanical ventilation was required, and medical insurance enrollment.

#### 2.3.2 Post-traumatic growth

PTG was assessed using the Chinese version of the Post-Traumatic Growth Inventory (PTGI). Developed by Tedeschi et al. in 1996 (8) and revised by Chinese scholars (23), the PTGI comprises 20 items across five dimensions: appreciation of life, personal strength, new possibilities, relating to others, and spiritual change. It utilizes a 6-point Likert scoring method, with total scores ranging from 0 to 100. Higher scores indicate a higher level of PTG. Outcomes are categorized into low (0–59), moderate (60–65), and high (66–100) levels of growth (24). The Cronbach’s *α* coefficient for the total scale in this study was 0.93.

#### 2.3.3 Resilience

Resilience was evaluated using the Chinese version of the Connor-Davidson Resilience Scale (CD-RISC), revised by Chinese scholars in 2007 (25). It comprises 25 items across three dimensions–tenacity, strength, and optimism–rated on a scale from 0 (“not true at all”) to 5 (“true nearly all the time”). The total score ranges from 0 to 100 points, with higher scores indicating better psychological resilience. The Cronbach’s *α* coefficient for the total scale in this study was 0.92.

#### 2.3.4 Perceived social support

Perceived social support was evaluated using the Chinese version of the Perceived Social Support Scale (PSSS), complied by Jiang et al. (26). The PSSS includes 12 items across three dimensions: family support, friend support, and other support. It employs a 7-point Likert scale, with total scores ranging from 12 to 84 points. Higher scores reflect stronger perceived social support. The Cronbach’s *α* coefficient for the total scale in this study was 0.91.

#### 2.3.5 Coping style

The Simple Coping Style Questionnaire, compiled by Xie et al. (27), was used to evaluate individual coping styles; it comprises 20 items across two dimensions: positive coping and negative coping. Scores for positive coping total 36 points, while those for negative coping totals 24 points, rated on a four-point Likert scale. If the average score for the positive coping dimension exceeds that of the negative coping dimension, it suggests that respondents primarily employ positive coping methods, and vice versa. In this study, the Cronbach’s *α* coefficient was 0.78 for the positive coping subscale and 0.70 for the negative coping subscale.

### 2.4 Data collection

Data were collected from the NICUs of the participating hospitals in Fujian Province between January 2022 and December 2022. Prior to the survey, researchers established a WeChat group for training guide nurses or primary nurses across the participating hospitals. The survey’s purpose, participant selection criteria, and questionnaire completion methods were communicated through this group. A questionnaire QR code was also disseminated via the group, which nurses then printed onto cards. Guide nurses or primary nurses instructed participants to complete the survey when they visited the hospital to provide breast milk or inquire about their child’s condition. Each parent was allowed to fill out the questionnaires once. Incomplete or missing data resulted in the disqualification of the submission.

### 2.5 Data analysis

Data were exported from the questionnaire platform and analyzed using SAS 9.4. A two-sided *p*-value of <0.05 was considered statistically significant. Descriptive statistics were used to describe general information as well as PTG, resilience, perceived social support, and coping style scores. Tolerance and the variance inflation factor were utilized to assess the multicollinearity between the total scores for resilience, perceived social support, and coping style. Pearson correlations were performed to identify the correlations between parents’ PTG, psychological resilience, perceived social support, and coping styles.

AMOS 24.0 was utilized to construct the structural equation model for the influencing factors of PTG and to analyze the mechanisms of different influencing factors on PTG. The following criteria were used to assess the model’s fitness: adjusted goodness-of-fit index (AGFI) > 0.80, comparative fit index (CFI) > 0.90, goodness-of-fit index (GFI) > 0.80, incremental fit index (IFI) > 0.90, normed fit index (NFI) > 0.90, root mean square error of approximation (RMSEA) < 0.08, Tucker-Lewis’s index (TLI) > 0.90, and the chi-square/degrees of freedom ratio (*χ*2/df) < 3.00 (28). A 95% bootstrapping confidence interval (CI) was used to evaluate the significance of the indirect and direct effects of the model.

## 3 Results

### 3.1 General information and characteristics of VLBW infants’ parents

In this study, 360 questionnaires were distributed across nine hospitals in Fujian Province, including one secondary and eight tertiary hospitals. After excluding questionnaires with inconsistent answers or those with straight-line markings, 344 valid questionnaires were included in the analysis, yielding an effective response rate of 95.56%. Most participants were fathers (*n* = 207, 60.17%). A slightly higher proportion of mothers experienced pregnancy complications (*n* = 216, 62.79%). The average gestational age of the infants was 30.33 ± 1.86 weeks. The median birth weight was 1315.00 g with an interquartile range of 242.50 g, and approximately 97.38% of the infants required mechanical ventilation. Results of the univariate analysis indicated that educational level, conception method, and maternal pregnancy complications were influencing factors of PTG (all *p* < 0.05). Additional demographic data and the distribution of PTG are presented in Table 1.

**Table 1:** Comparison of demographic and PTG scores of parents with VLBW infants (*n* = 344).

Variables	Categories	*n* (%)	PTG (mean ± SD)	*t*/*F*	*p*
Relation to child	Father	207(60.17)	47.98 ± 12.55	0.130	0.900
	Mother	137(39.83)	47.80 ± 13.09		
Occupation	Cadres and staff	118 (34.30)	48.26 ± 13.34	0.230	0.878
	Farmers, workers	66 (19.19)	47.71 ± 13.12		
	Freelance work	135 (39.24)	48.05 ± 12.17		
	Unemployed	25 (7.27)	46.00 ± 12.52		
Education	High school or below	107 (31.10)	45.77 ± 13.17	3.670	0.027
	Bachelor or associate	211 (61.34)	48.39 ± 12.09		
	Master’s degree or above	26 (7.56)	52.85 ± 14.85		
Age (years)	≦35	263 (76.45)	47.88 ± 12.78	−0.080	0.934
	>35	81 (23.55)	48.01 ± 12.71		
Residence	Rural	86 (25.00)	46.64 ± 12.21	−1.070	0.287
	Town	258 (75.00)	48.33 ± 12.92		
Monthly income per capita (yuan)	<5,000	72 (20.93)	48.00 ± 13.06	0.330	0.722
	5,000–7,999	127 (36.92)	47.22 ± 12.41		
	≥8,000	145 (42.15)	48.47 ± 12.94		
Religious belief	Have	42 (12.21)	45.02 ± 12.12	1.570	0.118
	None	302 (87.79)	48.31 ± 12.80		
Child gender	Male	194 (56)	47.76 ± 12.47	−0.250	0.802
	Female	150 (43.60)	48.11 ± 13.14		
Delivery mode	Natural delivery	142 (41.28)	47.15 ± 14.04	−0.890	0.373
	Caesarean section	202 (58.72)	48.44 ± 11.76		
Mode of conception	Natural conception	294 (85.47)	47.02 ± 12.96	−3.790	0.001
	Assisted insemination	50 (14.53)	53.12 ± 10.03		
Fetus number	Single birth	273 (79.36)	47.71 ± 13.14	−0.560	0.578
	Twins/multiple births	71 (20.64)	48.66 ± 11.18		
Parity	First child	184 (53.49)	47.86 ± 13.00	−0.080	0.937
	Second child and above	160 (46.51)	47.97 ± 12.49		
Complications of pregnancy	Yes	216 (62.79)	49.25 ± 12.76	−2.540	0.011
	No	128 (37.21)	45.66 ± 12.45		
Accept mechanical ventilation or not	Yes	335 (97.38)	48.01 ± 12.75	0.850	0.395
	No	9 (2.62)	44.33 ± 12.78		
Enroll in medical insurance or not	Yes	187 (54.36)	48.27 ± 13.06	−0.570	0.571
	No	157 (45.64)	47.48 ± 12.39		
Fetal age^*^		344	30.33 ± 1.86		
Birth weight^#^		344	1315.00 (242.50)		

* Mean ± standard deviation.

# Median (interquartile).

### 3.2 Resilience, perceived social support, and positive coping were positively associated with PTG

Table 2 shows the means and standard deviations of the variables. The total PTGI score was 47.91 ± 12.75, with the highest scores observed in the “appreciation of life” dimension (2.56 ± 0.71) and the lowest in the “new possibilities” dimension (2.27 ± 0.75). The total CD-RISC score was 54.62 ± 11.75, with the “strength” dimension scoring the highest (2.23 ± 0.51). The total PSSS score was 66.11 ± 9.09, with “family support” scoring the highest (5.65 ± 0.85). The mean scores for positive and negative coping styles were 2.05 ± 0.40 and 1.17 ± 0.50, with the positive coping scores being overall higher than negative coping scores, indicating that most participants positive coping. There was no co-linear relationship between the resilience, perceived social support, and coping styles, with tolerance values exceeding 0.1 and variance inflation factor values below 5 (Additional file 2).

**Table 2:** The mean values and standard deviations of the scores for the PTGI, CD-RISC, PSSS, and SCSQ (*n* = 344).

Variables	Mean ± SD
PTGI (total score: 0–100)	47.91 ± 12.75
PTGI (potential point: 0–5)	2.40 ± 0.64
appreciation of life	2.56 ± 0.71
personal strength	2.44 ± 0.75
new possibility	2.27 ± 0.75
relating to others	2.32 ± 0.76
spiritual change	2.31 ± 0.79
CD-RISC (total score: 0–100)	54.62 ± 11.75
CD-RISC (potential point: 0–4)	2.18 ± 0.47
tenacity	2.16 ± 0.52
strength	2.23 ± 0.51
optimism	2.16 ± 0.50
PSSS (total score: 12–84)	66.11 ± 9.09
PSSS (potential point: 1–7)	5.51 ± 0.76
family support	5.65 ± 0.85
friend support	5.38 ± 0.87
other support	5.50 ± 0.86
Positive coping (potential point: 0–3)	2.05 ± 0.40
Negative coping (potential point: 0–3)	1.17 ± 0.50

PTGI indicates Post-traumatic Growth Inventory, used to measure the individual’s level of post-traumatic growth; CD-RISC indicates the Connor-Davidson Resilience Scale, used to measure the individual’s level of psychological resilience; PSSS indicates the Perceived Social Support Scale, used to measure the individual’s level of perceived social support.

Pearson correlation analysis was conducted to assess the relationships between parents’ PTG, psychological resilience, perceived social support, and coping styles across various dimensions (Table 3). Results indicated that the scores for PTGI, CD-RISC, PSSS, and positive coping were positively inter-correlated (all *p* < 0.05). Notably, PTGI and its constituent factors were negatively correlated with negative coping style scores. Meanwhile, psychological resilience was positively correlated with perceived social support and positive coping style scores (all *p* < 0.01). Additionally, perceived social support was positive correlated with positive coping (*p* < 0.01). These findings support H1.

**Table 3:** Results of Pearson’s correlation analysis for PTGI, CD-RISC, PSSS, and SCSQ (*r* value).

Variables	PTGI1	PTGI2	PTGI3	PTGI4	PTGI5	PTGI	CD-RISC1	CD-RISC2	CD-RISC3	CD-RISC	PSSS1	PSSS2	PSSS3	PSSS	SCSQ1	SCSQ2
PTGI1	1															
PTGI2	0.74^**^	1														
PTGI3	0.71^**^	0.70^**^	1													
PTGI4	0.58^**^	0.55^**^	0.61^**^	1												
PTGI5	0.75^**^	0.62^**^	0.61^**^	0.54^**^	1											
PTGI	0.92^**^	0.84^**^	0.86^**^	0.75^**^	0.85^**^	1										
CD-RISC1	0.77^**^	0.71^**^	0.72^**^	0.62^**^	0.69^**^	0.84^**^	1									
CD-RISC2	0.80^**^	0.67^**^	0.71^**^	0.60^**^	0.68^**^	0.83^**^	0.80^**^	1								
CD-RISC3	0.64^**^	0.57^**^	0.61^**^	0.53^**^	0.61^**^	0.70^**^	0.62^**^	0.65^**^	1							
CD-RISC	0.83^**^	0.74^**^	0.77^**^	0.66^**^	0.74^**^	0.89^**^	0.96^**^	0.92^**^	0.75^**^	1						
PSSS1	0.37^**^	0.41^**^	0.37^**^	0.42^**^	0.32^**^	0.44^**^	0.40^**^	0.40^**^	0.34^**^	0.42^**^	1					
PSSS2	0.37^**^	0.44^**^	0.44^**^	0.47^**^	0.34^**^	0.47^**^	0.45^**^	0.44^**^	0.39^**^	0.47^**^	0.62^**^	1				
PSSS3	0.35^**^	0.40^**^	0.38^**^	0.43^**^	0.31^**^	0.43^**^	0.39^**^	0.40^**^	0.32^**^	0.42^**^	0.66^**^	0.72^**^	1			
PSSS	0.41^**^	0.47^**^	0.45^**^	0.50^**^	0.37^**^	0.51^**^	0.47^**^	0.47^**^	0.40^**^	0.50^**^	0.86^**^	0.89^**^	0.90^**^	1		
SCSQ1	0.45^**^	0.45^**^	0.46^**^	0.44^**^	0.38^**^	0.51^**^	0.43^**^	0.44^**^	0.33^**^	0.46^**^	0.37^**^	0.44^**^	0.37^**^	0.44^**^	1	
SCSQ2	−0.11^*^	−0.10	−0.09	−0.02	−0.13^*^	−0.11^*^	−0.10	−0.09	−0.14^**^	−0.11^*^	−0.14^*^	−0.05	0.01	−0.07	−0.04	1

(1) PTGI, the scores of Post-traumatic Growth Inventory; PTGI 1–5, factor score of “Appreciation of Life,” “Personal Strength,” “New Possibilities,” “Relating to Others,” “Spiritual Change.” (2) CD-RISC, the scores of Connor-Davidson Resilience Scale; CD-RISC 1–3: factor score of “Tenacity,” “Strength,” “Optimism.” (3) PSSS, the scores of Perceived Social Support Scale; PSSS 1–3, factor score of “Family Support,” “Friend Support,” “Other Support.” (4) SCSQ, the scores of Simplified Coping Style Questionnaire; SCSQ1-2, factor score of “Positive Coping, “Negative Coping”.

* Significance *p* < 0.05.

** Significance *p* < 0.01.

### 3.3 Resilience can directly affect PTG and can also indirectly affect PTG through the mediating role of positive coping

**Figure 1:**
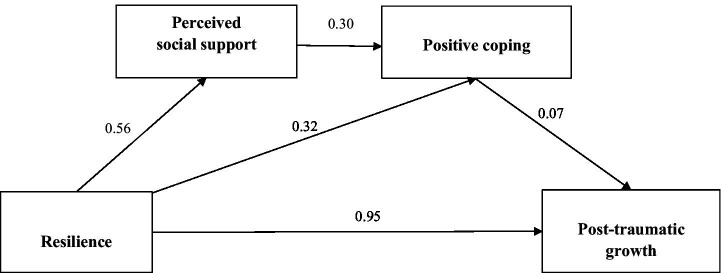
Structural equation modeling results. The structural model has adequate fit to the data. All the coefficients in this figure are standardized and significant at level 0.05.

Figure 1 illustrates the mediation model, depicting the mediating effects of perceived social support and positive coping on the relationship between psychological resilience and PTG. With psychological resilience as the independent variable and PTG as the dependent variable, perceived social support and positive coping were utilized as intermediate variables within the structural equation model to test the hypothesized relationships. The path coefficients for resilience and PTG, resilience and positive coping, resilience and perceived social support, perceived social support and positive coping, and positive coping and PTG were 0.95, 0.32, 0.56, 0.30 and 0.07, respectively (*p* < 0.05). The model exhibited a favorable fit index (CFI = 0.98, TLI = 0.98, IFI = 0.98, NFI = 0.97, GFI = 0.95, AGFI = 0.93, RMSEA = 0.05, and CMIN/DF = 1.97).

The specific effect values for each path within the model are detailed in Table 4. H2 is therefore is partially supported, indicating that psychological resilience has a positive overall effect on PTG, with the total effect of psychological resilience on PTG being 1.657 (95%CI = 1.452–1.877), a direct effect of 1.598 (95%CI = 1.388–1.824). This suggested that the influence of psychological resilience on PTG is predominantly direct.

**Table 4:** The total effects, direct effects, and indirect effects of every path in this model.

Estimate	*β*	95%CI			Relative effect (%)
		Lower	Upper	*p*	
Total effects					
Resilience → post-traumatic growth	1.657	1.452	1.877	<0.001	
Perceived social support → post-traumatic growth	0.018	0.003	0.042	0.015	
Direct effects					
Resilience → post-traumatic growth	1.598	1.388	1.824	<0.001	96.4
Resilience → positive coping	1.039	0.611	1.489	<0.001	65.9
Indirect effects					
Resilience → positive coping → post-traumatic growth	0.039	0.009	0.079	0.014	2.4
Resilience → perceived social support → positive coping → post-traumatic growth	0.020	0.004	0.047	0.014	1.2
Resilience → perceived social support → positive coping	0.539	0.301	0.837	<0.001	34.1
Perceived social support → positive coping → post-traumatic growth	0.018	0.003	0.042	0.015	100

### 3.4 Mediating effects of perceived social support and positive coping in the relationship between resilience and PTG

Indirect effects were tested using the bias-corrected bootstrap method with 5,000 resamples. Table 4 shows the indirect effects of psychological resilience and PTG. Results show that H3 is supported. Perceived social support and positive coping styles served as chain mediators between psychological resilience and PTG (effect = 0.020, 95%CI = 0.004–0.047), indicating that social support does not play a complete mediating role in PTG. Instead, it can only exert an indirect effect on the PTG of parents of VLBW infants through their coping styles.

## 4 Discussion

We evaluated how resilience affects the PTG of parents of VLBW infants and examined the mediating effects of perceived social support and positive coping. The mediation model’s results confirmed that resilience significantly contributes to PTG, either directly or indirectly. The mean PTG score for parents was low, indicating that most parents (77.62%) exhibited low levels of PTG. The findings support the positive correlation between the resilience and PTG of parents of VLBW infants. Resilience can directly influence PTG and can also indirectly influence it indirectly through the mediating role of positive coping. Interestingly, a serial mediation pathway between resilience and PTG through perceived social support and positive coping style was also identified.

In a previous study utilizing the same measurement tool (29), the mean PTG score of parents of NICU-hospitalized premature infants was higher (61.89 ± 17.89) than that in the current study. The lower PTG scores observed in this study may be attributed to the emotional and physical stress experienced by parents of VLBW infants. The stress of the NICU environment, the uncertainty of VLBW infants’ prognosis, and the financial burden of treatment can all contribute to parents’ emotional and physical exhaustion. Parents’ psychological states may be particularly affected owing to the severity of premature birth, and leading to a slower PTG process (30). Moreover, this study measured variables 4–6 weeks after the infants’ hospitalization, whereas prior studies measured these variables several months to a year after a child’s hospitalization or discharge (7, 31). The timing of outcome measurement may explain the differences in findings. PTG is a psychological phenomenon that occurs following adversity, and it may not be immediately evident when the trauma first occurs; rather, the growth occurs gradually over time (11). The low PTG scores in this study suggest that parents have not effectively coped with the stress and challenges of their infants’ illness after 4–6 weeks of hospitalization. Professional counseling and psychological intervention are necessary to enhance the resilience levels of Chinese parents of VLBW preterm infants, thereby facilitating their PTG.

This study identifies resilience as a protective factor for parents in the current context, consistent with the notion that resilient individuals exhibit greater PTG following stressful events (32). However, some researchers argue that because resilient individuals are not severely impacted by traumatic situations, they may not experience PTG; this assertion requires further investigation (33). The path analysis results from the structural equation model in this study demonstrate that the direct effect of psychological resilience on PTG is greater than its indirect effect, indicating the predominance of the direct effect. Therefore, resilience-oriented interventions should be employed to enhance parents PTG, including mindfulness-based cognitive therapy (34), cognitive-behavioral therapy (35), and dialectical behavioral therapy (36).

Resilience is a psychological process that enables individuals to adapt to adverse circumstances through positive coping strategies. Prior research has shown that positive coping styles have a significant impact on PTG (14). True PTG is the realization of constructive cognition or the materialization of fantasies through behavior. By seeking emotional support, taking constructive action, and employing other positive coping strategies, individuals can reduce the perceived impact of traumatic events (37). This suggests that parents who utilize positive coping strategies are more likely to experience PTG following their infants’ illnesses.

Based on the SCA model, PTG model, and the relevant literature, we constructed a hypothetical model and verified its fitness and path significance. Perceived social support and positive coping can indirectly influence PTG. Perceived social support is positively correlated with PTG, indicating that it is an essential factor in promoting PTG among parents of VLBW infants. Parents with a robust support network may be better equipped to handle the stress of their infants’ illnesses, leading to higher levels of PTG. However, social support influenced PTG only indirectly in our study. This result differed from previous findings (16), which could be attributed to the fact that PTG can be facilitated by internalizing perceived external social support and transforming it into a positive coping skill. The findings of this research imply that healthcare providers should focus not only on enhancing the social support systems of parents of VLBW infants but also on parents’ methods of coping with their children’s illnesses.

## 5 Limitations

This study has several limitations. First, the period of data collection was short and only one parent per family was invited to participate in the study. These conditions may have introduced selection bias, affecting the generalizability of the findings. Second, infant disease status was not incorporated into the analysis. These factors should be addressed by future research. Finally, the 4–6 week window of this study may have only captured the initial stage of PTG or “potential growth tendency” rather than fully developed PTG. Future studies should extend the follow-up duration to verify the stability of PTG. Moreover, PTG is influenced by various factors, but this study only explored its correlation with psychological resilience, perceived social support, and coping style. The relationship with a broader range of variables should be investigated in future research.

## 6 Conclusion

This study found that most (77.62%) parents with VLBW infants exhibit low levels of PTG, a phenomenon that warrants active attention from clinical staff. The PTG of these parents is positively associated with their psychological resilience, perceived social support, and positive coping. Psychological resilience can directly and indirectly influence PTG through the mediating roles of perceived social support and positive coping. This finding underscores the necessity for clinical nurses and related personnel to actively seek ways to enhance parents’ psychological resilience, perceived social support, and positive coping strategies and effectively foster their ongoing PTG.

## Data Availability

The original contributions presented in the study are included in the article/supplementary material, further inquiries can be directed to the corresponding author.
