# Supplementary material for: Post-traumatic growth, resilience, perceived social support, and coping style among parents of very low birth weight infants: a multi-center, cross-sectional study
Source: Front Public Health. 2025 Dec 4;13:1686820. doi: 10.3389/fpubh.2025.1686820 (PMC12711482; doi:10.3389/fpubh.2025.1686820)
Supplement: Supplementary file 1 [file Presentation_1.pdf]

Survey Questionnaire

Dear Participants：

We are currently conducting a survey on the post-traumatic growth of parents with very low birth weight infants, aiming to understand whether you have achieved a certain degree of post-traumatic growth after experiencing the traumatic event of your baby being born prematurely and admitted to the neonatal intensive care unit. We will assess your general information, post-traumatic growth, coping methods, and social support to provide effective intervention measures in the future, to alleviate anxiety, maintain physical and mental health, and promote the recovery of the child.

Please answer according to your actual situation. There is no correct answer to all questions. They will be strictly confidential and only used for academic research. Thank you for your cooperation and support, wishing you a happy life!

(I) Parent and Patient General Information Survey Form

1.Your relationship with the patient: ☐Father ☐ Mother

2.Your occupation: ☐ Cadre,Staff(Civil servant/Teacher/Soldier/Medical staff) ☐ Farmer ☐ Worker ☐ Freelance ☐ Housework (Unemployed)

3.Your level of education: ☐ Junior high school and below ☐ High school or technical secondary school Bachelor's or Associate degree ☐ Master's degree or above

4.Your age:______years old

5.Your place of residence: ☐ Rural ☐ Urban

6.Household per capita monthly income: ☐ 1000-2999 yuan ☐ 3000-4999 yuan ☐ 5000-7999 yuan ☐ ≥8000 yuan

7.Your religious belief: ☐ None ☐ Yes

8.Baby's gender: ☐ Male ☐ Female

9.Delivery method: ☐ Vaginal delivery ☐ Cesarean section

10.Conception method: ☐Natural conception ☐Artificial assisted fertilization

11.Number of fetuses in this pregnancy: ☐ Single ☐ Twins ☐ Multiples

12.This pregnancy is the: ☐ First ☐ Second ☐ Third ☐ Fourth or more

13.Maternal pregnancy complications:☐Gestational diabetes ☐ Premature rupture of membranes ☐ Preeclampsia, gestational diabetes ☐ Placental abruption ☐ Other ☐ None

14.Did the baby receive respiratory support with a ventilator during hospitalization: ☐ Yes ☐ No

15.Source of the baby's medical expenses: ☐ Self-funded ☐ Commercial insurance ☐ Medical insurance ☐ Commercial insurance and medical insurance ☐ Rural cooperative medical care

16.Baby's gestational age at birth:_____weeks

17.Baby's birth weight:______g
