# Supplementary material for: Post-traumatic growth, resilience, perceived social support, and coping style among parents of very low birth weight infants: a multi-center, cross-sectional study
Source: Front Public Health. 2025 Dec 4;13:1686820. doi: 10.3389/fpubh.2025.1686820 (PMC12711482; doi:10.3389/fpubh.2025.1686820)
Supplement: Supplementary file 2 [file Presentation_2.pdf]

Collinearity diagnosis between variables

Variables	t	P	Tolerance	VIF
Resilience	27.77	0.00	0.68	1.48
Perceived social support	1.76	0.08	0.69	1.44
Positive coping	4.15	0.00	0.73	1.37
Negative coping	-0.47	0.64	0.99	1.01
